# Development, reliability and validity of the Safe Use of Mobility Aids Checklist (SUMAC) for 4-wheeled walker use in people living with dementia

**DOI:** 10.1186/s12877-020-01865-5

**Published:** 2020-11-11

**Authors:** Susan W. Hunter, Alison Divine, Humberto Omana, Ed Madou, Jeffrey Holmes

**Affiliations:** 1 School of Physical Therapy, University of Western Ontario, Room 1588, Elborn College, London, ON N6G 1H1 Canada; 2 Faculty of Biological Sciences, Sport and Exercise Science, University of Leeds, Leeds, England; 3 Faculty of Health and Rehabilitation Sciences, University of Western Ontario, London, Ontario Canada; 4 School of Occupational Therapy, University of Western Ontario, London, Ontario Canada

**Keywords:** Dementia, Walkers, Geriatric assessment, Reliability, Validity

## Abstract

**Background:**

Balance and gait problems are common and progressive in dementia. Use of a mobility aid provides physical support and confidence. Yet, mobility aid use in people with dementia increases falls three-fold. An assessment tool of mobility aid safety in people with dementia does not currently exist. The objectives of this study were: 1) to develop a tool for the evaluation of physical function and safe use of a 4-wheeled walker in people with dementia, and 2) to evaluate its construct and criterion validity, inter-rater and test-retest reliability and minimal detectable change.

**Methods:**

Healthcare professionals (HCP) experienced in rehabilitation of people with dementia participated in focus groups for item generation of the new tool, The Safe Use of Mobility Aid Checklist (SUMAC). The SUMAC evaluates physical function (PF) and safe use of the equipment (EQ) on nine tasks of daily life. Reliability was evaluated by HCP (*n* = 5) scored participant videos of people with dementia (*n* = 10) using a 4-wheeled walker performing the SUMAC. Inter-rater and test-retest reliability was assessed using intra-class correlation coefficients (ICC). Construct validity evaluated scores of the HCPs to a consensus HCP panel using Spearman’s rank-order correlations. Criterion validity evaluated SUMAC-PF to the Performance-Oriented Mobility Assessment (POMA) gait subscale using Spearman’s rank-order correlations.

**Results:**

Three focus groups (*n* = 17) generated a tool comprised of nine tasks and the components within each task for physical function and safe use. Inter-rater reliability was statistically significant for SUMAC-PF (ICC = 0.92, 95%CI (0.81, 0.98), *p* < 0.001) and SUMAC-EQ. (ICC = 0.82, 95%CI (0.54, 0.95), *p* < 0.001). Test-retest reliability was statistically significant for SUMAC-PF (ICC = 0.89, 95%CI (0.81, 0.94), *p* < 0.001) and SUMAC-EQ. (ICC = 0.88, 95%CI (0.79, 0.93), *p* < 0.001). As hypothesized, the POMA gait subscale correlated strongly with the SUMAC-PF (r_s_ = 0.84), but not EQ (r_s_ = 0.39).

**Conclusions:**

The focus groups and research team developed a tool of nine tasks with evaluation on physical function and safe use of a 4-wheeled walker for people with dementia. The SUMAC tool has demonstrated content validity for the whole scale and good construct and criterion validity for the SUMAC-PF and SUMAC-EQ. The subscores of the SUMAC demonstrated excellent to good inter-rater and test-retest reliability.

**Supplementary Information:**

The online version contains supplementary material available at 10.1186/s12877-020-01865-5.

## Background

People with dementia have an annual falls risk of 60–80%, twice that of their cognitively healthy aged peers [1], and have a higher risk of major fall-related injuries, such as hip fractures [2]. Falls in people with dementia are multifactorial, including orthostatic syncope from disease-related changes in autonomic function, prescription medications, vision problems, functional status and the severity of the disease [3, 4]. Among adults with dementia, balance problems and gait disorders are common and progressive [5, 6]. These balance and gait problems are associated with an increased risk of falls in people with dementia [4, 7]. The provision of a mobility aid (e.g., cane or walker) can be an important treatment option to compensate for balance and gait impairments [8] as mobility is fundamental to successful aging and quality of life in older adults [9].

Use of a mobility aid has been found to improve walking stability [8, 10] and allow greater ambulation and social participation in cognitively healthy older adults [11]. Yet, use of a mobility aid in people with dementia has been found to be independently associated with a three-fold increased odds of falling [4]. This finding has serious implications for long-term care facilities as 79% of residents have cognitive impairment, 70% use a 4-wheeled walker and 80% fall each year [9, 12, 13].

The increased risk of falls with use of a mobility aid is multifactorial, factors include uncompensated physical deficits, increased cognitive demands, and cognitive deficits related to insight and memory [4, 14–16]. Use of a mobility aid is a complex motor activity requiring motor sequencing and coordination, navigating and selecting a path through in the environment, along with remembering strategies for use (e.g., use of the brakes). The transition to use of a mobility aid is an important milestone for people with dementia as it can occur when brain function may be challenged to accommodate greater resource utilization required to use the mobility aid [15, 16]. Additionally, cognitive impairments may compromise learning of new tasks resulting in unsafe practices when using the equipment [8].

The majority of older adults obtain a mobility aid without consulting a healthcare professional [17, 18]. In addition, there are accessibility issues for people using 4-wheeled walkers. Lindemann et al. identified obstacle clearance and opening a door against the direction of walking as the biggest problem for older adults using a 4-wheeled walker [19]. In the absence of professional input, the mobility aid may not appropriately compensate for deficits in balance and gait, and the person with dementia will not receive training on safe use of the equipment. Research by Hunter et al. [20] found that caregivers of people with dementia were the key people to identify the need for a mobility aid, obtained the mobility aid, and provided ongoing reminders for its use in daily activities to the person with dementia.

Rehabilitation can improve the functional mobility of people with dementia through gains in strength, balance and endurance [21]. Yet access to rehabilitation is not guaranteed and even those who do receive services may have ongoing deficits after completion of an exercise program. Therefore, prescription of mobility aids is still an important clinical tool to compensate for deficits. People with dementia newly learning or experienced in using a 4-wheeled walker demonstrate increased cognitive demands and a deterioration in gait in distracting situations and when manoeuvring around obstacles [15, 22]. Additionally, the uptake and safe use of mobility aids may be impacted by cognitive impairment through a lack of self-awareness or incomplete learning to use the equipment safely [8]. The appropriate use of mobility aids is important for falls prevention, yet incorrect equipment use is common in older adults [23]. Fall prevention strategies that are successful in older adults without cognitive problems have not been successful in reducing fall risk in people with dementia. As a result of insufficient evidence, the most prominent fall prevention guidelines do not provide any recommendations for people with cognitive impairment [24].

Currently, there is no standardized assessment scale to evaluate function and the safe use of a 4-wheeled walker. A standardized scale would facilitate reporting among healthcare professionals, care planning to optimize client safety and targeted interventions to lessen falls risk. Additionally, it is important for delivery of care to monitor change in function over time as a consequence of progression of cognitive deficits or participation in rehabilitation. Therefore, the objectives of this study were: 1) to develop a tool for the evaluation of physical function and safety with use of a 4-wheeled walker in people with dementia, and 2) to evaluate its construct and criterion validity, inter-rater and test-retest reliability and minimal detectable change.

## Methods

This project involved two phases: Phase 1 was the item generation and development of the assessment scale (cross-sectional design) and Phase 2 was a reliability and validation study for the scale developed in Phase 1 (cross-sectional design).

### Phase 1: item generation and development

Items were generated from two sources for content validity: i) the research team and ii) healthcare professionals. The research team (SWH, AD, JH) generated items from reviewing existing mobility scales, discussion with experts in mobility among people with dementia and a search of healthcare textbooks on clinical skills for mobility aid training. We also conducted two focus groups of healthcare professionals from relevant disciplines in London, Ontario Canada providing care across health settings for people with dementia (*n* = 12; 1 geriatrician, 2 registered nurses, 5 physical therapists and 4 occupational therapists). The healthcare professionals were working in the areas of acute hospital care, community care, long-term care, day hospital and rehabilitation hospital. Eleven (92%) participants had 10 or more years of total clinical experience working in geriatrics. All healthcare professionals provided written informed consent prior to the start of data collection.

Our focus groups identified the 4-wheeled walker as the most common mobility aid used in this patient population. Therefore, items were sought that represented 1) the minimum functional tasks needed to assess and evaluate independence and safety with the use of a 4-wheeled walker, and 2) the components within each task that can be objectively evaluated with respect to function and safe use.

The focus groups and work by the research team generated 11 tasks and components within each task. To render the scale manageable in the clinical and research setting, the authors sought to reduce the number of items. The information was presented to an independent panel of five healthcare professionals (1 registered nurse, 2 physical therapist, and 2 occupational therapists) with more than 10 years of clinical experience in geriatrics and who had not participated in the first set of focus groups. The participants were asked, “Rank how important it is to include each task, regardless of setting, when assessing physical function and safety with a 4-wheeled walker”. The 11 identified tasks were ranked on a 5-point Likert scale with a score of 1 representing “least important” and 5 representing “most important”. The scores for each task were summed and averaged across raters. Tasks were kept if the average score was rated ≥4, indicative of the item being rated as either important or most important (Table 1).

**Table 1:** Results of ranking tasks generated in focus groups to be considered part of a minimum set of tasks that every person should complete, regardless of living setting, when assessing physical function and safety with a 4-wheeled walker

Task	Scores on 5-point Likert scale	
	Median Score	Range of Scores
Sit to stand	5	3–5
Pivot turn and sit in a chair	5	4–5
Walking on a level surface	5	3–5
Walking with horizontal head turns	4.5	3–5
Walking with concurrent cognitive task	4	4–5
Walking around obstacles (figure of 8)	5	All scored at 5
Walking up a ramp	3	2–5
Walking down a ramp	3	2–5
Walking through an open doorway	5	1–5
Open, walk through and close door that opens away	4	2–5
Open, walk through and close door that opens in	4	2–5

5-point Likert scale with a score of 1 representing “least important” and 5 representing “most important”

The evaluation resulted in a final selection of nine tasks with task components that comprise The Safe Use of Mobility Aids Checklist (SUMAC). The research team categorized the components in each task into two separate areas of focus, physical function (PF) which includes items related to an individual’s physical ability to perform the task (e.g., stand independently) and use of the equipment (EQ) which includes items related to an individual’s safety in using their 4-wheeled walker (e.g., brakes engaged on walker). Items for PF and EQ are not equally present across the tasks and reflect to some extent the difficulty of the activity. The distribution of scoring items for each task (number of PF items:number of EQ items) are: sit to stand (3:7); pivot turn to sit in chair (3:7); walking on level surface (11:6); walking with horizontal head turns (1:6); walking with concurrent cognitive task (1:6); walking around obstacles – figure of eight (1:6); walk through an open doorway (1:5); open, walk through and close door that opens away from the person (1:10); and open, walk through and close door that opens in to person (1:10). Physical function items are rated on a 3-point scale (0, 1, 2) and use of equipment is a dichotomous scale, where the item is scored as observed yes (1) or no (0). Total PF scores can range from 0 to 40 and the total EQ score can range from 0 to 63. A higher score in each category indicates better physical function and safety. (The full scale is available in the Supplementary File 1 and at www.mobility-in-aging-lab.ca).

### Phase 2: reliability and validity evaluation

This phase of the study involved the recruitment of people living with dementia and a separate sample of health care professionals. The project was conducted in accordance to the Declaration of Helsinki and was approved by the University of Western Ontario Research Ethics Board for Health Sciences Research Involving Human Subjects.

People with dementia who used a 4-wheeled walker for ambulation were recruited from a local day program to be evaluated with the SUMAC. Participants had a diagnosis of probable AD from a geriatrician based on the National Institute of Neurologic and Communicative Disorders and Stroke-AD and Related Disorders Association (NINCDS-ARDRA) criteria [25]. Inclusion criteria were: 50 years of age and older, English proficiency, able to follow instructions, mild to moderate disease severity, able to walk 60 m without support from another person and have a substitute decision maker (in all cases a family member of the person with dementia) to provide information about health and daily activities. Exclusion criteria were any muscle and/or nerve problem that limited movement. Informed written consent was provided by either the participant or their substitute decision maker and then the participant provided assent to participate in the study.

Demographic and clinical information collected on the participants included age, gender, falls in the previous 12 months, number of prescription medications, number of comorbidities and instrumental activities of daily living, as per the Lawton-Brody Instrumental Activities of Daily Living and Basic Activities of Daily Living scales [26]. Participants also completed the Iconographical-Falls Efficacy Scale, which has been validated in older adults with cognitive impairment [27]. Participants (*n* = 10) were videotaped while performing the nine tasks of the SUMAC.

The raters were a convenience sample of five healthcare professionals (5 physiotherapists). All healthcare professionals provided written informed consent prior to the start of data collection. The healthcare professionals were working in the areas of acute hospital care, community care, and long-term care. Two people (40%) had 10 or more years of total clinical experience working in geriatrics. The inclusion criterion was: registered healthcare professional with experience working with older adults with dementia. Each assessor attended a one-hour one-on-one training session on the use of the SUMAC. In the training session, each person was presented with a description of the development of the tool and the rationale for its creation, received a copy of the assessment tool and was given a detailed instruction of the components and items within the tool. The last activity in the training session was evaluating videos of a person performing the nine tasks of the SUMAC.

#### Reliability

In the reliability evaluation, each of the healthcare professionals was asked to view each participant’s video and evaluate them using the SUMAC on two occasions. The two visits were set 1 week apart. In both visits the viewing order of each participant’s video was randomized, but all components of the SUMAC were presented together and in the order in the tool for each participant.

#### Validity

The evaluation of construct validity was performed by a panel of eight healthcare professionals with clinical experience working with older adults and people with dementia (6 physical therapists, 2 registered kinesiologists) who had not participated in any aspect of the development or reliability evaluations of the SUMAC. All members of the panel had the same training session used in the reliability study. The panel viewed the participant videos in a group setting in a single session with discussion until consensus was reached in scoring the physical function and safe use of the equipment. The scores of the HCP panel were compared to the scores of the individual HCP from the reliability study.

Our sample of five HCP who participated in the reliability study of the SUMAC also completed the gait subscale of the Performance-Oriented Mobility Assessment (POMA) gait subscale on each participant. The HCP viewed videos of each participant walking at their self-selected usual gait speed using their 4-wheeled walker over a distance of a 6-m. Criterion validity was evaluated by comparing the PF and EQ scores of the SUMAC against the Performance-Oriented Mobility Assessment (POMA) gait subscale scores [28]. The POMA is a reliable tool in people with dementia [29].

### Data analysis

#### Reliability

Values for relative and absolute reliability were calculated. An a priori sample size calculation (α = 0.05 and β = 0.20) for the reliability study indicated that 10 participants and 5 assessors making 2 evaluations would be needed if a target ICC of 0.90 was desired [30]. This sample size minimized recruitment and participant burden while optimizing the use of multiple healthcare professionals as assessors of the videos.

The relative reliability values of inter-rater and test-retest reliability were calculated for the PF and EQ domain scores of the SUMAC using the intra-class correlation coefficient (ICC). Repeated measurements by different raters on the same day were used to calculate inter-rater reliability, while repeated measurements by the same rater on different days were used to calculate test-retest reliability. The ICC values were categorized to provide a means to quantify the strength of the relationship; therefore an ICC value greater than 0.90 was considered excellent, between 0.80 to 0.89 was good, 0.70 to 0.79 was fair, and values less than 0.70 are considered of questionable clinical value [31].

Two measures of absolute reliability were calculated: standard error of measurement (SEM) and minimal detectible change with a 95% confidence interval (MDC_95_) for the PF and EQ domain scores of the SUMAC. The SEM is the measurement error associated with a single value and is expressed in the same units as the scale [31]. The smaller the SEM, the greater the absolute reliability. The MDC95 is an estimate of the smallest change in the score that can be detected beyond measurement error [32]. It is also measured in the same units as the measurement scale. For the present study, the SEM was calculated using pooled standard deviation (SD) and ICC values for each group. Calculations of SEM and MDC_95_ were:

$$ SEM= SD\sqrt{\left(1- ICC\right)},{MDC}_{95}= SEM\times \sqrt{2}\times 1.96. $$

#### Validity

Spearman’s rank-order correlations were also used to assess construct validity between each healthcare professional and the separate HCP panel consensus scores. Three scores were compared: each participant’s mean score for each of the nine tasks and a total score using the minimum and maximum scores.

Spearman’ rank-order correlation analysis was used to evaluate criterion validity between the gait component of the POMA, and the SUMAC-PF and SUMAC-EQ scores. We hypothesized that the POMA score would be moderately correlated with the SUMAC-PF score and not correlated with the SUMCA-EQ score.

To interpret Spearman’s correlation coefficients related to the assessment of criterion and construct validity, the following thresholds were used: ≥0.50 was deemed strong, 0.31–0.49 was moderate to strong, 0.11–0.30 was weak to moderate, and ≤ 0.10 was considered a non-existent relationship [33]. All statistical analyses were performed using SPSS version 25.0 (IBM Inc., Chicago, IL, USA). Statistical significance was set at *p* < 0.05.

## Results

The demographic characteristics of the ten people with dementia who were evaluated in the reliability phase of the study are presented in Table 2.

**Table 2:** Characteristics of people with dementia who participated in the reliability study for the SUMAC. (*n* = 10)

Variable	Mean ± SD, or n (%)
Age (years)	88.5 ± 4.2
Gender, n (% female)	6 (60.0%)
Instrumental Activities of Daily Living	0.8 ± 0.9
Basic Activities of Daily Living	3.8 ± 1.1
History of Falls in the Past 12 Months, n (%)	1 (10.0%)
Fear of Falling, n (%)	3 (30.0%)
Number of Prescription Medications	7.2 ± 3.7
Number of Comorbidities	2.9 ± 1.1

*SD* Standard deviation

### Reliability

The values for the absolute reliability were the following: SEM for the PF was 1.31 and the EQ was 1.93; the MDC_95_ was 3.62 for PF and 5.35 for EQ. A good to excellent inter-rater reliability was observed for both testing sessions in the PF and EQ domains (Table 3) Additionally, good test-retest reliability was observed for the PF (ICC = 0.89, 95%CI (0.81 to 0.94), *p* < 0.001) and EQ. (ICC = 0.88, 95%CI (0.79 to 0.93), *p* < 0.001) domains.

**Table 3:** Scores and reliability values for the two components of the SUMAC

	Component scores of the Safe Use of Mobility Aids Checklist	
	Physical Function	Interaction with Equipment
**Mean (SD), range**		
Assessment #1	31.25 (3.81), 23–39	44.75 (5.40), 28–55
Assessment #2	29.80 (4.10), 18–37	43.46 (5.75), 28–53
**Relative Reliability (Intraclass correlation coefficients (95%CI),** ***p*-value)**		
Assessment #1: Inter-rater reliability	0.72 (0.31, 0.92), *p* = 0.003	0.84 (0.61, 0.96), *p* < 0.001
Assessment #2: Inter-rater reliability	0.92 (0.81, 0.98), *p* < 0.001	0.82 (0.54, 0.95), *p* < 0.001
Test-retest reliability	0.89 (0.81, 0.94), *p* < 0.001	0.88 (0.79, 0.93), *p* < 0.001
**Absolute Reliability**		
Standard Error of Measurement (SEM)	1.31	1.93
Minimum Detectable Change (MDC_95_)	3.64	5.35

*SD* Standard deviation, *CI* Confidence interval

### Validity

**Fig. 1:**
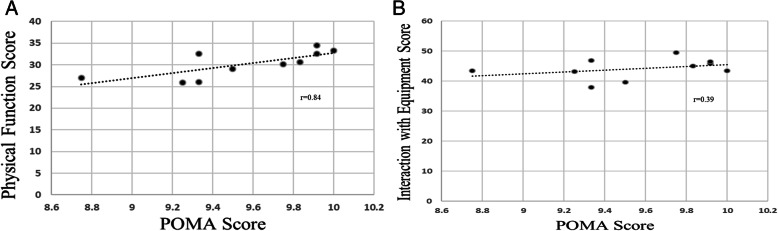
Construct validity as measured through Spearman’s correlation analysis of SUMAC physical performance scores (**a**) and interaction with equipment scores (**b**) to scores of the Performance-Oriented Mobility Assessment (POMA) among healthcare practitioners

In the evaluation of construct validity, moderate to strong positive correlations for the PF score (mean total score: r_s_ = 0.92, *p* < 0.001; minimum score: r_s_ = 0.93, *p* < 0.001; maximum score: r_s_ = 0.68, *p* = 0.03) and EQ score (mean total score: r_s_ = 0.82, *p* = 0.004; minimum score: r_s_ = 0.60, *p* = 0.06; maximum score: r_s_ = 0.90, *p* = 0.03) (Fig. 1a and b).

**Fig. 2:**
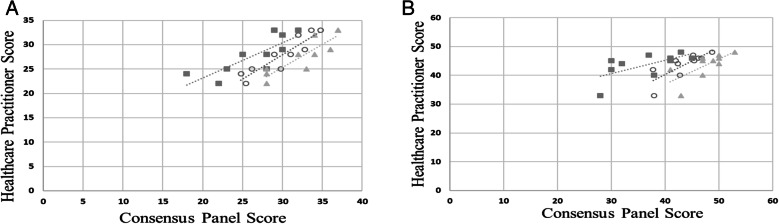
Criterion validity as measured through Spearman’s correlation analysis of mean, minimum and maximum scores of individual healthcare practitioners (HCP) and consensus scores of healthcare professionals for physical function (PF) (**a**) and safe use of the equipment (EQ) (**b**) domains of the SUMAC

In the evaluation of criterion validity, a strong positive correlation was observed between the POMA gait scores and PF scores (r_s_ = 0.84) (Fig. 2a and b). A weak positive correlation was observed between the POMA gait scores and EQ scores (r_s_ = 0.39).

## Discussion

This study reports the development and psychometric properties of a new tool for the assessment of physical function and safe use of a 4-wheeled walker for people with dementia. In the scale development phase, content validity of the items in the scale was achieved using input from researchers in the field of geriatrics and focus groups of healthcare professionals with expertise in the care of people with dementia. The research team and focus groups generated an assessment tool comprised of nine tasks. Within each task is a list of items that clinicians would rate for physical function and safe use. The assessment tool, the Safe Use of Mobility Aids Checklist (SUMAC), yields scores in physical function and safe use of the equipment in each task. There was strong support for construct and criterion validity of the SUMAC tool. The SUMAC was determined to have good to excellent inter-rater and test-retest reliability, with an MDC_95_ of 3.62 for PF and 5.35 for EQ. Overall, the psychometric properties provide support for the use of the scale in clinical practice.

Our focus groups clearly indicated that in their clinical practice 4-wheeled walkers were the most commonly prescribed and used mobility aid for this population. This observation is consistent with reports that the rate of walker use has greatly increased in recent years and that 4-wheeled walkers are the most common prescribed mobility aid for older adults [34]. There is also a strong link between 4-wheeled walker use and fall-related injuries among older adults who use a mobility aid. Stevens et al. [35] found use of walkers was associated with seven times as many injuries compared to use of canes. Van Riel et al. [36] found that most injuries sustained while using a 4-wheeled walker resulted from a fall, 60% of these injuries were fractures and hip fracture (25%) was the most common. The three-fold increased odds of falls in people with dementia over the cognitively normal, as well as the former group’s higher risk of sustaining major fall-related injuries, warrants standardizing an assessment tool for healthcare professionals to identify physical function and safe use of a 4-wheeled walker.

Our criterion validity analysis demonstrated that an observational gait assessment with the POMA gait subscale was correlated with the PF score of the SUMAC, but not with the EQ score. This relationship is consistent with our hypothesis because physical strength, balance and quality of gait are independent of unsafe techniques for use of the equipment. A study by Robinovitch et al. [37] found that 74% of long-term care residents were classified as habitual users of assistive devices and that a quarter of falls occurred while using an assistive device. Specifically, there was an increased risk of falls when transferring to use the mobility aid and the researchers identified safety concerns of people not appropriately using the prescribed device [37]. Our assessment tool includes the assessment of transitions between sitting and standing, and the challenges identified by Lindemann et al. [19] of manoeuvring the walker when navigating doors that open away and towards the user of the device.

Balance decreases with progression of cognitive impairment in dementia [38]. Postural control is affected in many ways by dementia; in particular, there are visual perceptual changes, slower sensory processing, reduced motor responses and increased attentional demands that impact static and dynamic balance [39]. A person with dementia commencing use of a 4-wheeled walker after diagnosis presents unique challenges for rehabilitation professionals. It is important to recognize that people with dementia have a preserved capacity for learning [40]. Training protocols that use procedural (implicit) learning optimize acquisition and retention of new skills [39]. There is some evidence to suggest that these methods are clinically useful in assisting those with dementia to learn and retain the skills for proper use of their walker [41]. Reinforcement of instructions by all healthcare members facilitates the process of procedural learning. The SUMAC scale presents a standardized list, in sequential order, of activities to be completed within the nine tasks. The tool has the potential to facilitate uniform expectations of performance with the equipment and education with appropriate reinforcement for people using a 4-wheeled walker.

There are several strengths of the study that we would like to highlight. Input was provided by a range of clinicians, healthcare professionals, and academics with expertise in geriatrics and dementia. We have provided a comprehensive evaluation of validity and psychometric properties of reliability. Additionally, the use of functional tasks while using the 4-wheeled walker is an advantage to people with dementia. This is the first tool to allow clinicians to objectively quantify, standardize, and track progress of an individual’s ability to safely use a mobility aid. There are some limitations that should be noted. The SUMAC was created solely for the assessment of 4-wheeled walker use and thus the tool is not applicable to canes or crutches. In our reliability phase of our study, inclusion criteria of participants was restricted to those with mild to moderate disease severity. Future research should examine the psychometric properties of the SUMAC in people with more advanced disease severity as it is not expected that performance of the tool will be comparable in severe disease. While we had an appropriate sample size to achieve power for the analysis, further evaluation should be conducted with a larger sample using in-person evaluation of performance.

## Conclusion

The focus groups and research team created an assessment tool, the SUMAC, comprising nine tasks and components within each task to evaluate physical function and safe use of a 4-wheeled walker by people with dementia. The SUMAC demonstrated good to excellent inter-rater and test retest reliability, as well as strong support for construct and criterion validity. Therefore, the SUMAC shows promise as a new tool to assess function and mobility aid safety in a population in which falls and mobility aid use are prevalent. Future research should examine the psychometric properties of the SUMAC in people with more advanced disease severity and evaluate its use in conjunction with interventions to improve gait in people with dementia using a 4-wheeled walker.

## Supplementary Information

**Additional file 1.** Safe Use of Mobility Aids Checklist (SUMAC).

## Data Availability

The datasets used and/or analyzed during the current study are available from the corresponding author on reasonable request.

## Abbreviations

HCP: Health care professional
ICC: Intra-class correlation coefficient
MDC_95_: Minimal Detectable Difference
POMA: Performance-Oriented Mobility Assessment tool
SEM: Standard error of measurement
SUMAC: Safe Use of Mobility Aids Checklist tool
SUMAC-PF: Physical Function component of the Safe Use of Mobility Aids Checklist
SUMAC-EQ: Equipment use component of the Safe Use of Mobility Aids Checklist
