# Supplementary material for: Development, reliability and validity of the Safe Use of Mobility Aids Checklist (SUMAC) for 4-wheeled walker use in people living with dementia
Source: BMC Geriatr. 2020 Nov 11;20:468. doi: 10.1186/s12877-020-01865-5 (PMC7659047; doi:10.1186/s12877-020-01865-5)
Supplement: Supplementary file 1 — Additional file 1. Safe Use of Mobility Aids Checklist (SUMAC). [file 12877_2020_1865_MOESM1_ESM.docx]

**S**afe **U**se of **M**obility **A**id **C**hecklist **- SUMAC** (v1.2e_2020)

**TASK 1: TRANSITION OF SIT TO STAND**

**Testing procedure**: People will start from a seated position in a standard height chair with armrests. On the command, “Go”, they will stand, walk forward on a level surface for 6 meters at a self-selected comfortable pace, turn around, walk back to the chair, turn and sit back down. (Evaluation of Task 1 and 2 are combined in the same assessment)

Equipment: Standard height chair with arm rests

| **Physical Function** | | | | **Interaction with equipment** | | |
| --- | --- | --- | --- | --- | --- | --- |
|  | **Scoring** | | |  | **Scoring** | |
|  | **0** | **1** | **2** |  | **0** | **1** |
| **Transition Sit to Stand** | | | | | | |
| **Sit to stand from chair (single attempt)** | Unable | Able, uses arms to help | Independent, no use of arms | Walker positioned directly in front and within arm’s reach |  |  |
| **Sit to stand attempts** | Unable to do independently | Able, but requires >1 attempt | Able, only requires 1 attempt | Brakes engaged on walker |  |  |
| **Immediate balance upon standing (first 5 seconds)** | Unsteady, loses balance or near loss | Unsteady, uses arms for support | Steady without arm support | Reaches one hand at a time to grasp handle of walker as stands |  |  |
|  |  |  |  | Feet remain within base of support of walker |  |  |
|  |  |  |  | Upright posture of trunk |  |  |
|  |  |  |  | Establishes balance before unlocking walker |  |  |
|  |  |  |  | Cueing (reverse score, yes=0, no=1)  [At any time during the task] |  |  |
| **Comments** | | | | **Comments** | | |

**Physical Function Score Task 1: ____________ Interaction with Equipment Score Task 1: _____________ (Higher scores represent better function)**

**TASK 2: GAIT WITH PIVOT TURN TO APPROACH CHAIR TO SIT**

**Testing procedure**: People will start from a seated position in a standard height chair with armrests. On the command, “Go”, they will stand, walk forward on a level surface for 6 meters at a self-selected comfortable pace, turn around, walk back to the chair, turn and sit back down. (Evaluation of Task 1 and 2 are combined in the same assessment)

Equipment: Standard height chair with arm rests

| **Physical Function** | | | | **Interaction with equipment** | | |
| --- | --- | --- | --- | --- | --- | --- |
|  | **Scoring** | | |  | **Scoring** | |
|  | **0** | **1** | **2** |  | **0** | **1** |
| **Gait with pivot turn to approach chair to sit** | | | | | | |
| **Gait with turn so properly aligned to chair to sit down** | Unable without physical assistance or does not align self to chair independently | Turns slowly, several small steps to catch balance following turn | Independent, steady with no loss of balance and continuous steps | Feet remain within base of support of walker |  |  |
| **Walk backwards so feels chair behind legs** | Unable without physical assistance or does not walk backwards independently | Able but slow, discontinuous steps and unsteady | Independent, steady with no loss of balance and continuous steps | Maintains hands of hand grips of walker during turn |  |  |
| **Stand to sit** | Misjudged distance or body alignment or falls into chair | Uses arms or not a smooth motion | Smooth controlled lowering into chair without use of arms | Maintains upright posture |  |  |
|  |  |  |  | Feet do not collide with walker |  |  |
|  |  |  |  | Brakes engaged on walker prior to starting to sit |  |  |
|  |  |  |  | Reaches one hand at a time to grasp arm of chair as sits |  |  |
|  |  |  |  | Cueing (reverse score, yes=0, no=1)  [At any time during the task] |  |  |
| **Comments** | | | | **Comments** | | |

**Physical Function Score Task 2: ____________ Interaction with Equipment Score Task 2: _____________ (Higher scores represent better function)**

**TASK 3: AMBULATION ON A LEVEL SURFACE**

**Testing Procedure**: People will start from a standing position and then will walk on a level surface at a self-selected comfortable pace for up to 60 meters.

Equipment: None

| **Physical Function** | | | | **Interaction with equipment** | | |
| --- | --- | --- | --- | --- | --- | --- |
|  | **Scoring** | | |  | **Scoring** | |
|  | **0** | **1** | **2** |  | **0** | **1** |
| **Ambulation on level surface** | | | | | | |
| **Initiation of gait** | Hesitancy or multiple attempts | No hesitancy |  | Feet remain within base of support |  |  |
| **Step length-right** | Right foot does not pass left stance foot (step to gait) | Right foot passes left stance foot (step through gait) |  | Feet do not collide with walker |  |  |
| **Step height-right** | Right foot does not completely clear the floor | Right foot completely clears floor |  | Maintains upright posture |  |  |
| **Step length-left** | Left foot does not pass right stance foot (step to gait) | Left foot passes right stance foot (step through gait) |  | Equipment does not collide with environmental obstacles |  |  |
| **Step height-left** | Left foot does not completely clear the floor | Left foot completely clears floor |  | Maintains control of equipment |  |  |
| **Step symmetry** | Left step does not equal right | Left equals right |  | Cueing (reverse score, yes=0, no=1)  [At any time during the task] |  |  |
| **Step continuity** | Stopping or discontinuity between steps | Steps appear continuous |  |  |  |  |
| **Path** | Marked deviation from straight path | Mild to moderate deviation from straight path | No deviations from straight path |  |  |  |
| **Trunk** | Marked sway | No sway, but flexion of trunk and/or knees | No sway, no flexion of trunk or knees |  |  |  |
| **Walking distance (without rest)** | Unable  <5 m | 5-30m  30-50m | >50m |  |  |  |
| **Walking independence** | > minimum physical assist | Minimum physical assist of 1 person  Supervision | Independent |  |  |  |
| **Comments** | | | | **Comments** | | |

**Physical Function Score Task 3: ____________ Interaction with Equipment Score Task 3: _____________ (Higher scores represent better function)**

**TASK 4: WALKING WHILE PERFORMING TASK OF HORIZONTAL HEAD TURNS**

**Testing Procedure:** People will walk at a self-selected walking pace on a level surface for 6 meters and turn around an obstacle and walk back 6 meters while turning their head from side to side.

Equipment: One orange cone to be placed on the floor.

| **Physical Function** | | | | **Interaction with equipment** | | |
| --- | --- | --- | --- | --- | --- | --- |
|  | **Scoring** | | |  | **Scoring** | |
|  | **0** | **1** | **2** |  | **0** | **1** |
| **Walking while performing task of horizontal head turns** | | | | | | |
|  | Major decrease in gait speed, staggers/loses balance, or stops | Minor decrease to gait speed within smooth gait path | Performs head turns smoothly with no change in gait | Feet remain within base of support of walker |  |  |
|  |  |  |  | Feet do not collide with walker |  |  |
|  |  |  |  | Maintains upright posture |  |  |
|  |  |  |  | Walker does not make contact with obstacle |  |  |
|  |  |  |  | Maintains control of equipment |  |  |
|  |  |  |  | Cueing (reverse score, yes=0, no=1)  [At any time during the task] |  |  |
| **Sum** |  |  |  | **Sum** |  |  |
| **Comments** | | | | **Comments** | | |

**Physical Function Score Task 4: ____________ Interaction with Equipment Score Task 4: _____________ (Higher scores represent better function)**

**TASK 5: WALKING WHILE PERFORMING A CONCURRENT COGNITIVE TASK**

**Testing Procedure**: People will walk at their self-selected walking pace on a level surface for 6 meters and turn around an obstacle and walk back 6 meters while performing a concurrent cognitive task aloud. The difficulty of the cognitive task is individualized to each person’s abilities.

Equipment: One orange cone to be placed on the floor.

| **Physical Function** | | | | | **Interaction with equipment** | | |
| --- | --- | --- | --- | --- | --- | --- | --- |
|  | **Scoring** | | | |  | **Scoring** | |
|  | **0** | **1** | **2** | **3** |  | **0** | **1** |
| **Walking while performing a concurrent cognitive task** | | | | | | | |
| **Cognitive task: _____________________________________** | | | | | | | |
|  | Unable to do both at the same time, stops walking (posture second) | Unable to do both at the same time, stops secondary task (posture first) | Able to do both at the same time, slowing of gait or talking, minor unsteadiness | Able to do both at same time without change in gait or talking, no unsteadiness | Feet remain within base of support of walker |  |  |
|  |  |  |  |  | Feet do not collide with walker |  |  |
|  |  |  |  |  | Maintains upright posture |  |  |
|  |  |  |  |  | Walker does not make contact with obstacle |  |  |
|  |  |  |  |  | Maintains control of equipment |  |  |
|  |  |  |  |  | Cueing (reverse score, yes=0, no=1)  [At any time during the task] |  |  |
| **Sum** |  |  |  |  | **Sum** |  |  |
| **Comments** | | | | | **Comments** | | |

**Physical Function Score Task 5: ____________ Interaction with Equipment Score Task 5: _____________ (Higher scores represent better function)**

**TASK 6: WALKING WHILE NEGOTIATING OBSTACLES**

**Testing Procedure**: People will walk in a figure of eight pattern. Two cones will be placed 1.5 meters apart and people will maneuver around the cones to complete a figure of eight. The person will go around the figure of eight path twice for a single test session.

Equipment: Two orange cones to be placed on the floor.

| **Physical Function** | | | | **Interaction with equipment** | | |
| --- | --- | --- | --- | --- | --- | --- |
|  | **Scoring** | | |  | **Scoring** | |
|  | **0** | **1** | **2** |  | **0** | **1** |
| **Walking while negotiating obstacles** | | | | | | |
|  | Unable to clear obstacles or requires physical assistance | Able to do with slowing of gait speed and change in steps | Able to do without changing gait speed and no unsteadiness | Feet remain within base of support of walker |  |  |
|  |  |  |  | Feet do not collide with walker |  |  |
|  |  |  |  | Maintains upright posture |  |  |
|  |  |  |  | Maintains control of equipment |  |  |
|  |  |  |  | Walker does not make contact with obstacle |  |  |
|  |  |  |  | Cueing (reverse score, yes=0, no=1)  [At any time during the task] |  |  |
| **Sum** |  |  |  | **Sum** |  |  |
| **Comments** | | | | **Comments** | | |

**Physical Function Score Task 6: ____________ Interaction with Equipment Score Task 6: _____________ (Higher scores represent better function)**

**TASK 7: WALKING THROUGH AN OPEN DOORWAY**

**Testing procedure**: People will walk through an open doorway at their self-selected comfortable pace. There will be a 3 meter lead walk before the doorway to be able to judge for any alterations in gait.

Equipment: A standard single doorway opening.

| **Physical Function** | | | | **Interaction with equipment** | | |
| --- | --- | --- | --- | --- | --- | --- |
|  | **Scoring** | | |  | **Scoring** | |
|  | **0** | **1** | **2** |  | **0** | **1** |
| **Walking through an open doorway** | | | | | | |
|  | Major decrease in gait speed, staggers/loses balance, or stops | Minor decrease to gait speed within smooth gait path | No change in gait speed and smooth gait path | Feet remain within base of support of walker |  |  |
|  |  |  |  | Maintains hands of hand grips |  |  |
|  |  |  |  | Maintains upright posture |  |  |
|  |  |  |  | Walker does not make contact with doorframe |  |  |
|  |  |  |  | Cueing (reverse score, yes=0, no=1)  [At any time during the task] |  |  |
| **Sum** |  |  |  | **Sum** |  |  |
| **Comments** | | | | **Comments** | | |

**Physical Function Score Task 7: ____________ Interaction with Equipment Score Task 7: _____________ (Higher scores represent better function)**

**TASK 8: OPEN AND WALK THROUGH A DOOR THAT OPENS AWAY FROM PERSON**

**Testing Procedure**: Facing a door that opens away from the person, they will open the door, walk through it, and then close the door.

Equipment: Perform assessment with a door that does not have self-closing mechanism.

| **Physical Function** | | | | **Interaction with equipment** | | |
| --- | --- | --- | --- | --- | --- | --- |
|  | **Scoring** | | |  | **Scoring** | |
|  | **0** | **1** | **2** |  | **0** | **1** |
| **Open and walk through a door that opens away from the person** | | | | | | |
|  | Major decrease in gait speed, staggers/loses balance, stops | Minor decrease to gait speed within smooth gait path | No change in gait speed and smooth gait path | Approaches door at an angle so faces side of door that opens away (faces door knob side of door) |  |  |
|  |  |  |  | Reaches for door knob with hand closest to door and pushes door out without losing balance or tipping aid |  |  |
|  |  |  |  | Opens door wide enough to get body and mobility aid through opening |  |  |
|  |  |  |  | Both hands on hand grips of walker while walking through door way |  |  |
|  |  |  |  | Walks through doorway without colliding with doorframe or door with body or mobility aid |  |  |
|  |  |  |  | Once clear of door path, able to reach for door within arm’s reach to close it without colliding with body or mobility aid or needing to walk with one hand on walker |  |  |
|  |  |  |  | Feet remain within base of support of walker |  |  |
|  |  |  |  | Feet do not collide with equipment |  |  |
|  |  |  |  | Maintains upright posture |  |  |
|  |  |  |  | Cueing (reverse score, yes=0, no=1)  [At any time during the task] |  |  |
| **Sum** |  |  |  | **Sum** |  |  |
| **Comments** | | | | **Comments** | | |

**Physical Function Score Task 8: ____________ Interaction with Equipment Score Task 8: _____________ (Higher scores represent better function)**

**TASK 9: OPEN AND WALK THROUGH DOOR THAT OPENS TOWARDS THE PERSON**

**Testing Procedure**: Facing a door that opens towards from the person, they will open the door, walk through it, and then close the door.

Equipment: Perform assessment with a door that does not have self-closing mechanism.

| **Physical Function** | | | | **Interaction with equipment** | | |
| --- | --- | --- | --- | --- | --- | --- |
|  | **Scoring** | | |  | **Scoring** | |
|  | **0** | **1** | **2** |  | **0** | **1** |
| **Open and walk through door that opens towards the person** | | | | | | |
|  | Major decrease in gait speed, staggers/loses balance, stops | Minor decrease to gait speed within smooth gait path | No change in gait speed and smooth gait path | Approaches door at an angle so faces the hinges and body position is clear of door path |  |  |
|  |  |  |  | Reaches for door knob with hand closest to door and pulls door in without losing balance or tipping aid |  |  |
|  |  |  |  | Opens door wide enough to get body and mobility aid through opening |  |  |
|  |  |  |  | Both hands on hand grips of walker while walking through door way |  |  |
|  |  |  |  | Walks through doorway without colliding with doorframe/door with body or mobility aid |  |  |
|  |  |  |  | Once clear of door path, able to reach for door within arm’s reach to close it without colliding with body or mobility aid or needing to walk with one hand on walker |  |  |
|  |  |  |  | Feet remain within base of support of walker |  |  |
|  |  |  |  | Feet do not collide with equipment |  |  |
|  |  |  |  | Maintains upright posture |  |  |
|  |  |  |  | Cueing (reverse score, yes=0, no=1)  [At any time during the task] |  |  |
| **Sum** |  |  |  | **Sum** |  |  |
| **Comment** | | | | **Comment** | | |

**Physical Function Score Task 9: ____________ Interaction with Equipment Score Task 9: _____________ (Higher scores represent better function)**

**SUMAC** – **S**afe **U**se of **M**obility **A**id **C**hecklist

| **Task** | **Physical Function**  **(x/40)** | **Interaction with Equipment**  **(x/63)** |
| --- | --- | --- |
| TASK 1: TRANSITION OF SIT TO STAND |  |  |
| TASK 2: GAIT WITH PIVOT TURN TO APPROACH CHAIR TO SIT |  |  |
| TASK 3: AMBULATION ON A LEVEL SURFACE |  |  |
| TASK 4: WALKING WHILE PERFORMING TASK OF HORIZONTAL HEAD TURNS |  |  |
| TASK 5: WALKING WHILE PERFORMING A CONCURRENT COGNITIVE TASK |  |  |
| TASK 6: WALKING WHILE NEGOTIATING OBSTACLES |  |  |
| TASK 7: WALKING THROUGH AN OPEN DOORWAY |  |  |
| TASK 8: OPEN AND WALK THROUGH A DOOR THAT OPENS AWAY FROM PERSON |  |  |
| TASK 9: OPEN AND WALK THROUGH DOOR THAT OPENS TOWARDS THE PERSON |  |  |
| **TOTAL SCORE** |  |  |

**Note: Higher scores represent better function.**
